# Editorial: Understanding effective education: far transfer from a sociocultural and cognitive neural perspective

**DOI:** 10.3389/fpsyg.2025.1769420

**Published:** 2026-01-29

**Authors:** J. W. Whitlow, Paul Webb, Ines Meier

**Affiliations:** 1 Psychology Department, Rutgers University Camden, Camden, NJ, United States; 2 Faculty of Science, Nelson Mandela University, Port Elizabeth, South Africa

**Keywords:** educational interventions, far transfer, flexibility of approach, learning, relational mode

Webb and Whitlow (2019) suggested that there was considerable promise for understanding how to promote effective education by bringing together two different approaches to learning. One approach was a cognitive approach, derived from controlled experimentation on processes of learning and memory; the other approach was a sociocultural approach, derived from observations of communities of practice and the enculturation of knowledge. Despite their many differences, both approaches had come to share a view of effective education as a process that empowered learners to apply and adapt the knowledge they acquire in formal educational settings to solve problems and deal with situations that could be very different from those they were explicitly taught. In the language of transfer of training, both approaches had converged on the goal of promoting “far transfer.” With that goal, research efforts from both approaches need to identify paradigms that reliably produce such transfer, determine what common features those paradigms have, and develop theories that incorporate these features to create practical guidelines for educational implementations.

The papers in this Special Topic illustrate this mandate and show its potential to enhance education. They also suggest fruitful directions for research to explore the best ways for curricula to develop and sustain robust far transfer, whether at the molecular level of specific subject matter (e.g., Lai and Zhang; Mickey and McClelland), the molar level of general classroom instruction (seen in papers by Gamino et al., and Demetriou et al.), or at the theoretical level of essential elements of developmental and cognitive processes (seen in papers by Demetriou et al., Sofologi et al., Don et al., and Mickey et al.). Here we briefly summarize each contribution, then provide an overview of what we see as three integrating principles that emerge from these independent examples of effective education.

At the level of curricular materials, Lai and Zhang show benefits of dysfluency and signaling in multimedia materials for science education, and Mickey and McClelland show benefits of providing spatial representations for trigonometric functions, both for retention and for transfer. At the level of approaches to pedagogy, Demetriou et al. emphasize the need to align teaching focus with cognitive development, and Gamino et al. show that middle school teachers can enhance transfer by structuring their curricula around training executive function uses. Sofologi et al. offer some suggestive evidence for transfer across modalities (like from music training to reading) that draw on similar processing demands, as both reading and music training require integrating continuous sequences of elements. At a theoretical level, Don et al., Gamino et al., and Mickey et al. all emphasize in different ways the importance of students' learning robust forms of relational discovery.

## Three principles and a caveat

A first principle, recognized in all the papers, is that creating effective education requires attending to multiple factors, including students' motivation, students' and teachers' beliefs about intelligence, the structure of curricular scaffolding, and the nature of classroom environments. That effective education requires attention to multiple factors is, as a general point, not a revelation for educators and educational researchers. What we think we add to this general point is a focus on specific factors that promote “far transfer” so that activities that engage students and teachers also lead them to apply their knowledge to new situations.

A second principle is to help students think in a *relational mode*, such that they seek ways to relate one item of information to others in a meaningful way, whether by constructing arguments, adopting alternative perspectives or co-ordinating the relations among items in one context to relations among items in another context. One way to do this is through teaching skills of constructive argumentation that encourage taking alternative points of view; another way is to develop representational models that can generalize from one context to another.

A third principle is to encourage flexibility of thought and the use of multiple pathways to solve a problem or reach a goal. Rather than seeking a single framework with which to solve a given type of problem, learners need to be encouraged to accept that there can be multiple paths to reach a goal. This principle is seen particularly clearly in the work of Mickey et al., who make a strong case for students' use of both rules and the unit circle to solve trigonometry problems, for example. The importance of flexibility of representations has been noted before, as in Siegler's research (e.g., Siegler and Pyke, 2013) on the learning of fractions, but these papers reinforce that principle.

One caveat, articulated clearly in our series of papers by Akindipe, is that effective educational practices have to be judged not only by their impact on average performance by a class but also by their impact on individual students or groups of students. We note, for example, the recent analysis by Bouton et al. (2023) showing that educational techniques that work for most students are not effective for the weakest students without additional support.

Nonetheless, we are encouraged about the possibilities of making education more effective and promoting far transfer by combining insights from cognitive/neural approaches and sociocultural approaches. Further, the successes reported by Gamino et al. and Mickey et al. with implementations in actual classrooms demonstrate the approach is feasible. Combined with work like that of Bouton et al. (2023) and Reznitskaya and Wilkinson and their colleagues (Reznitskaya and Wilkinson, 2015; Wilkinson et al., 2017) showing success in training teachers to provide effective education, these papers suggest that new paradigms for teacher training and new forms of classroom instruction will enable our students not only to learn effectively and durably but also to apply their learning beyond the classroom.
